# The effect of particle properties on the depth profile of buoyant plastics in the ocean

**DOI:** 10.1038/srep33882

**Published:** 2016-10-10

**Authors:** Merel Kooi, Julia Reisser, Boyan Slat, Francesco F. Ferrari, Moritz S. Schmid, Serena Cunsolo, Roberto Brambini, Kimberly Noble, Lys-Anne Sirks, Theo E. W. Linders, Rosanna I. Schoeneich-Argent, Albert A. Koelmans

**Affiliations:** 1 The Ocean Cleanup Foundation, Martinus Nijhofflaan 2, 2624 ES Delft, The Netherlands; 2 Aquatic Ecology and Water Quality Management Group, Department of Environmental Sciences, Wageningen University & Research, P.O. Box 47, 6700 AA Wageningen, The Netherlands; 3 Takuvik Joint International Laboratory, Département de biologie et Québec-Océan, Université Laval, Quebec G1V 0A6, Canada; 4 Civil Engineering Department, Aalborg University, Fredrik Bajers Vei 5, 9100 Aalborg, Denmark; 5 Roger Williams University, Bristol, USA; 6 CABI Europe-Switzerland, Rue des Grillons 1, 2800 Delémont, Switzerland; 7 Carl von Ossietzky, University Oldenburg, ICBM-Terramare, Schleusenstr. 1, 26382 Wilhelmshaven, Germany; 8 IMARES - Institute for Marine Resources & Ecosystem Studies, Wageningen UR, P.O. Box 68, 1970 AB IJmuiden, The Netherlands

## Abstract

Most studies on buoyant microplastics in the marine environment rely on sea surface sampling. Consequently, microplastic amounts can be underestimated, as turbulence leads to vertical mixing. Models that correct for vertical mixing are based on limited data. In this study we report measurements of the depth profile of buoyant microplastics in the North Atlantic subtropical gyre, from 0 to 5 m depth. Microplastics were separated into size classes (0.5–1.5 and 1.5–5.0 mm) and types (‘fragments’ and ‘lines’), and associated with a sea state. Microplastic concentrations decreased exponentially with depth, with both sea state and particle properties affecting the steepness of the decrease. Concentrations approached zero within 5 m depth, indicating that most buoyant microplastics are present on or near the surface. Plastic rise velocities were also measured, and were found to differ significantly for different sizes and shapes. Our results suggest that (1) surface samplers such as manta trawls underestimate total buoyant microplastic amounts by a factor of 1.04–30.0 and (2) estimations of depth-integrated buoyant plastic concentrations should be done across different particle sizes and types. Our findings can assist with improving buoyant ocean plastic vertical mixing models, mass balance exercises, impact assessments and mitigation strategies.

Microplastics are defined as plastic particles smaller than 5 mm in length^1^. They can enter the marine environment either directly (e.g. via runoff or from aerosol deposition) or by fragmentation of larger plastic items already present in the ocean^1^. Buoyant microplastics are omnipresent in the marine environment^2^ and can act as transport vectors for rafting species^3^ and persistent organic pollutants^3^,^4^. They have also been found to be ingested by a variety of organisms, including those feeding at and below the ocean surface (e.g. whales^5^,^6^ and different fish species^7^,^8^). This can result in internal abrasion and blockage of digestive tracts^3^,^9^, as well as exposure to leaching chemicals^4^. To better understand the impact of microplastics on the marine environment, a thorough understanding of their distribution and contamination levels is needed^10^. Yet although buoyant microplastics are known to vertically mix in the water column^11^,^12^,^13^,^14^, most quantitative assessments of plastic debris have focused only on the sea surface^2^, sampling with manta (0–0.15 m) or neuston (0–0.25 m) nets. This is thought to have resulted in a significant underestimation of the total mass and number of buoyant plastics in the top layer of the oceans^11^,^12^,^15^, and calls for increased research on the vertical distribution of buoyant microplastics.

Several studies^10^,^16^,^17^,^18^,^19^ have corrected surface measurements of buoyant microplastics for vertical mixing using the hydrodynamic wind mixing model presented by Kukulka *et al*.^11^. This model predicts that the numerical abundance of buoyant microplastics in the mixed layer of the North Atlantic is at least 2.5 times greater than what is collected by neuston nets at the surface of this ocean^11^. Reisser *et al*.^12^ reported that up to 70% of the buoyant microplastics in the North Atlantic occur below the sea surface, depending on sea state and plastic characteristics^12^. A more recent and elaborate model, which includes Langmuir circulations and breaking waves, predicts that the abundance of buoyant microplastics in the subtropical gyres is 3.7 to 5.3 times higher than what is measured by neuston nets due to vertical mixing below 0.25 m^15^. However, all these models were evaluated with very limited data: the studies of Kukulka *et al*.^11^, Reisser *et al*.^12^ and Brunner *et al*.^15^ were based on 12, 12 and 21 multi-level net tows, respectively. Furthermore, different depth intervals were sampled: Kukulka *et al*.^11^ measured at 0, 5, 10 and 20 m depth, Brunner *et al*.^15^ took one surface and at least one subsurface sample (between 0 and 25 m depths), and Reisser *et al*.^12^ collected all plastics in the upper 5 m, at 0.5 m intervals^11^,^12^,^15^. Based on observations made over a limited range of sea states (<6 Beaufort state^11^,^12^,^15^), these authors predicted an abrupt decrease in buoyant microplastic concentrations from 0 to 3 m depth, where only few samples were available.

One of the main determinants of the vertical distribution of buoyant ocean plastics is the speed at which the particles rise to the surface once vertical downward mixing has occurred^11^. All studies mentioned above^11^,^12^,^15^ highlighted the key role of these rise velocities on the vertical distribution of microplastics under different sea states, acknowledging the effects of particle size, shape and density on the buoyant terminal rise velocity. Kukulka *et al*.^11^ only report an average buoyant terminal rise velocity from particles of unknown properties (i.e. shape, size and density); yet their model was used in other studies^10^,^16^,^17^,^18^,^19^ thereafter when correcting for vertical mixing. It is unknown for which plastic types and sizes the given buoyant terminal rise velocity is representative, and how a different buoyant terminal rise velocity might change the model outcomes. It is suggested that statistical distributions of plastic concentrations and buoyant rise velocities are required to improve these complex models^20^.

In this study, a large number of depth profiles for microplastics (0.5–5.0 mm) were measured over a range of sea states (Beaufort 1–5), as well as for different plastic types and size classes, using multi-level net tow samples (N = 47; depth: 0–5 m depth at 0.5 m intervals). We demonstrated that the vertical distribution of buoyant microplastics varies not only with environmental conditions, but also with the plastic size and shape. The buoyant terminal rise velocities of microplastics were measured, discriminating between different plastic sizes and types. The comprehensive datasets of microplastic terminal rise velocities and depth profiles reported in this study can be used to validate and improve hydrodynamic models predicting the vertical distribution of buoyant plastics at sea.

## Method

### Field sampling

**Figure 1:**
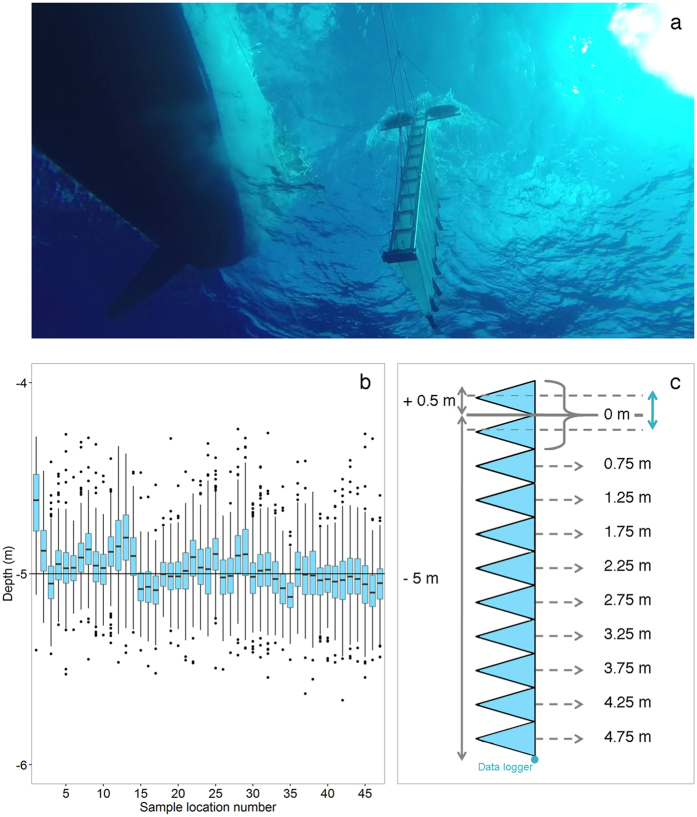
The multi-level trawl (MLT). (**a**) Underwater photograph of the MLT being towed; (**b**) boxplot of the depths recorded at the bottom of the MLT frame by a data logger (N = 47 MLT tows); (**c**) side view scheme of the MLT showing the inferred sampling depths of each net for a MLT tow under calm conditions.

A total of 47 net tows were carried out using a ‘multi-level trawl’ (MLT)^12^. The MLT is capable of sampling from the air-seawater interface up to a depth of 5 m, with 0.5 m intervals per net (net mesh: 330 μm; Fig. 1a). Sampling occurred between June 15 and July 9 2015 while sailing across the North Atlantic accumulation zone^21^, between 28° and 32° latitude and 38° and 60° longitude. Prior to each tow, a CastAway-CTD was deployed to profile the temperature and salinity of the local surface layer. Travelling at 1 to 2 knots, multi-level net tow durations ranged from 52 to 66 minutes, towing between 10:00 and 15:30 local time. Beaufort sea states^22^ were determined based on anemometer measurements taken regularly during each sampling period as well as visual observations of wave heights by the captain (over 20 years of sailing experience). With the continuous monitoring of wind and waves, we are confident to have obtained reliable estimates of Beaufort sea states for each trawl. Sample size differed per Beaufort scale, with N = 2 net tows for Beaufort 1, N = 9 for Beaufort 2, N = 11 for Beaufort 3, N = 22 for Beaufort 4 and N = 3 for Beaufort 5. After each tow, seawater was used to rinse each sample of the cod-end into a 150 μm sieve, before the sample content was transferred into aluminium bags which were kept frozen during the cruise and transportation to the laboratory. Detailed information related to these net tows (e.g. individual coordinates, metocean conditions, CTD profiles, sampling times and durations) can be found at Figshare^23^.

### Sample processing

The content of each cod-end was divided into two size classes by consecutively sieving the sample over 5000, 1500 and 500 microns meshes. The two size classes contained 0.5–1.5 mm and 1.5–5.0 mm particles. Since macroplastics (>5 mm) and plastics smaller than 0.5 mm were not the focus of this study, the materials in the 5000 micron sieve and the <0.5 mm particles were not analysed. The collected particles were placed in containers with filtered salt water (salinity 3.5%) to facilitate the separation of buoyant plastics from biomass. Floating particles identified as plastics were manually extracted using forceps, separated into types, and counted. Microplastics were classified as (1) ‘fragments’ - either hard plastic or plastic sheet fragments; (2) ‘lines’ – fibres from ropes, fishing nets or lines; (3) ‘foam’ – particles of expanded polystyrene (Styrofoam) or other types of foam material; and (4) ‘pellets’ – preproduction plastic nurdles in the shape of a cylinder, disk or sphere. Once counted and categorised, the particles were washed with distilled water, transferred to aluminium dishes, dried overnight at 60 °C, and weighed with an analytical balance (OHAUS EX324M, 0.1 mg readability).

### Depth profile analyses

Microplastic numerical/mass depth profiles were calculated by dividing the total number/mass of microplastic particles collected in each MLT net by the water volume that passed through the net. Depths sampled by each MLT net were inferred using the median depth recorded at the bottom of the MLT device by a HOBO U20L Water Level Data Logger (10 seconds, 0.1% accuracy; Fig. 1b): the deepest net (net 11) had a depth of 0.25 m above the median depth recorded by the data logger, while nets 10–1 had depths equal to the depth of the net below it minus 0.5 m (Fig. 1c). The median depth of all trawls combined was 5.0 m. The first MLT tow was the shallowest, with a median depth of 4.6 m, while MLT tow 35 was the deepest, with a median depth of 5.1 m. Water volumes filtered by the bottom 9 nets, which were constantly underwater, were calculated using net frame dimensions (0.3 × 0.5 m) and flowmeter rotations (1 rotation every 32 cm). The top net of the MLT device was mostly in the air, only sporadically filtering seawater due to wave-induced vertical movements. To compensate for this, samples from the top two nets were combined (depth = 0 m), with the calculated water volume varying with the median position of the air-water interface during each MLT tow. Based on the CTD data, the Mixed Layer Depth was calculated using the method of Kara *et al*. (2002), which is included in the rcalcofi package^24^.

**Figure 2:**
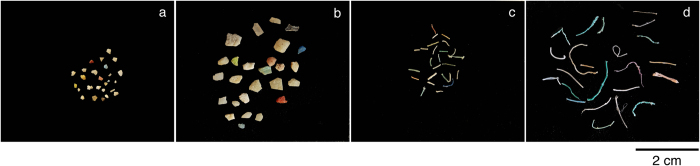
Microplastic sizes and types considered in the vertical distribution model. (**a**) Fragments 0.5–1.5 mm, (**b**) Fragments 1.5–5.0 mm, (**c**) Lines 0.5–1.5 mm and (**d**) Lines 1.5–5.0 mm.

Normalized microplastic concentrations were calculated by dividing the concentrations of each net sample by the surface concentration of that tow. This was done separately for each net tow using all line and fragment particles (0.5–5 mm), as well as separately for each plastic category: fragments 0.5–1.5 mm, fragments 1.5–5.0 mm, lines 0.5–1.5 mm and lines 1.5–5.0 mm (Fig. 2). Due to the rarity and unique physical properties of pellets and foams, these types of microplastics were not considered in our depth profile analyses.

### Buoyant terminal rise velocity experiments

The terminal rise velocities of microplastics were measured individually for the two sizes and types considered in this study, using the method described by Reisser *et al*.^12^. In this study, the term ‘buoyant terminal rise velocity’ refers to the constant rise velocity driven by the buoyancy of the particle in an undisturbed water column. One microplastic per category was selected from thirty randomly selected samples, resulting in 4 particles per sample. The length of these 120 particles was measured with a ruler and the particles were then soaked in filtered salt water (salinity 3.5%) for at least 4 hours. For each particle, the time to rise a distance of 16 cm inside a transparent cylinder (27 cm long, Ø 5 cm) filled with filtered salt water and closed airtight with a rubber stopper, was recorded in quadruplicates. The first 10 cm of the cylinder were regarded as the height necessary for the particles to stabilise and to reach its terminal velocity. In preliminary experiments it was tested that the buoyant terminal rise velocity was indeed reached within those 10 cm, as the particle did not accelerate in the remainder of the cylinder. The median value of the quadruplicates was calculated and afterwards used in a hydrodynamically parameterized model^11^.

### Vertical distribution model

An exponential decrease function was fitted to each normalized microplastic depth profile. In this model, the normalized microplastic concentration (N) was modelled as a function of the decrease coefficient (λ) and depth (z)^11^:
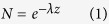


A lower λ value indicates stronger vertical mixing or a smaller buoyant terminal rise velocity. Due to some plastic concentrations at the surface being zero, resulting in “NA” values for normalized concentrations at greater depths, not all decrease coefficients could be calculated. Of the potential 188 λ values (47 stations *2 types *2 size classes), 181 could be calculated, both for mass and numerical concentrations. The parameter was fitted using the nls function from the nlstools package^25^ in R Studio^26^.

We tested whether Beaufort sea state, particle size and particle type affected λ values for mass and numerical profiles using a generalized linear mixed model (GLMM). The lme4 package in combination with the glmer test was used^27^, with the sampling location as a random effect, as four λ values were calculated for each location (for fragments 0.5–1.5 mm, fragments 1.5–5.0 mm, lines 0.5–1.5 mm, and lines 1.5–5.0 mm). Average λ values were fitted for numerical and mass concentrations, for each sea state (Beaufort 1–5). The 95% confidence interval of the statistical parameter was used for the uncertainty in the vertical distribution. The hydrodynamic implementation of the model of Kukulka *et al*.^11^ was compared to the fitted parameter λ, with λ = w_b_ (1.5 u_*w_ k H_s_)^−1^ 11. We refer to ‘hydrodynamic parameterization’ when the model of Kukulka *et al*.^11^ is used, and to ‘statistical parameterization’ when we used the fitted λ values. In this hydrodynamic parameterization, u_*w_ is the frictional velocity of water, k the von Karman constant, H_s_ the significant wave height and w_b_ the microplastic buoyant terminal rise velocity. The frictional velocity can be approximated as u_*w_ = 0.0012 W_10_, with W_10_ the wind speed at 10 m height^28^,^29^.

Different microplastic rise velocities were utilized when Kukulka’s model was applied. In addition to the mean ± standard deviation of the buoyant terminal rise velocity reported by Kukulka *et al*.^11^, results from the buoyant terminal rise velocity experiments of this study were also used. In the hydrodynamic parameterization, all plastic categories (two types and two size classes) were combined. The weighted mean buoyant terminal rise velocity was calculated separately for the mass and numerical distributions, because the relative contribution of each plastic category to the weighted mean differs between these distributions. Uncertainties of the weighted rise velocities were calculated using the weighted minimum and maximum rise velocities. When calculating the uncertainty interval of the vertical profiles predicted by this hydrodynamically parameterized model, both the uncertainties in the buoyant terminal rise velocity and in the Beaufort sea state were considered. Conservative uncertainty intervals were calculated by combining the weighted minimum/maximum rise velocities with the highest/lowest wind speeds associated with each Beaufort sea state. Wind speeds associated with the Beaufort scale were obtained from the UK Met Office^22^.

### Manta and neuston trawl underestimations

The microplastic amount missed by typical ocean surface samplers (i.e. manta and neuston nets) was estimated. For this underestimation (in %), equation (1) was integrated over depth. Plastic amounts captured by manta and neuston nets, up to a depth of 0–15 cm and 0–25 cm respectively, were compared to plastic amounts present in the first 5 m (sampling depth) and 17 m (average mixed layer depth of our MLT sampling period) of the water column. The underestimation percentages were calculated for all λ values, for numerical and mass concentrations, for all plastic categories and covering different sea states. Additionally, underestimations were calculated based on the hydrodynamic parameterization for the model of Kukulka *et al*.^11^, both using their average buoyant terminal rise velocity and different rise velocities per plastic category found in this study.

## Results

### Microplastic concentrations

**Figure 3:**
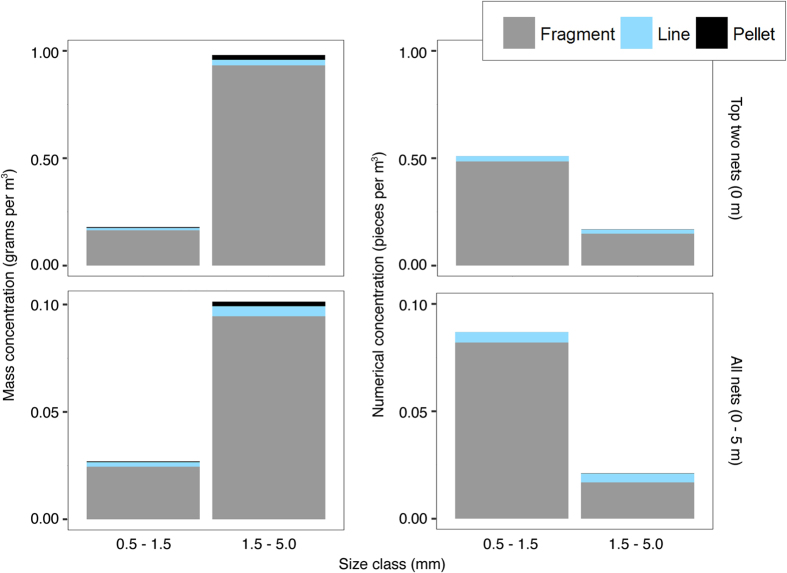
Mass and numerical concentrations of microplastics at 0 m and 0–5 m depth. Average microplastic mass concentrations (left) and numerical concentrations (right), for the ocean surface (top) and 0–5 m depth (bottom). Note the different y-scales for the different graphs. Foam concentrations cannot be visualised due to insufficient quantities.

In this study 29,850 microplastic particles were counted, with a total mass of 44.50 g. Of these particles, 27,045 were classified as fragments, 2,769 as lines, 33 as pellets and 3 as foam. Their total mass was 41.32 g, 3.00 g, 0.68 g and 0.6 mg, respectively. Mean buoyant microplastic concentrations decreased with depth (Fig. 3). For instance, on average there were 0.68 fragments m^−3^ and 1.13 mg m^−3^ of microplastic fragments at the ocean surface (top two nets) compared to 0.02 fragments m^−3^ and 6.1*10^−3^ mg m^−3^ in the bottom net. Depth integrated (0–5 m) microplastic mass concentrations (all plastic categories combined) were on average 9 times lower than surface concentrations (top two nets): 0.128 and 1.159 g m^−3^, respectively (Fig. 3). The difference was less pronounced for numerical concentrations: surface mean values were 0.68 particles m^−3^, while for the 0–5 m layer this was 0.11 particles m^−3^. Mass concentrations were higher for the 1.5–5.0 mm size class, while numerical concentrations were higher for the 0.5–1.5 mm size class. On average 337,000 particles km^−2^ were found in the upper 0.5 m of the water column, whereas the 5 m depth integrated average was 536,000 particles km^−2^. Depth integration of particle numbers, for the first 5 m only, increased microplastic numbers by almost 40%. The depth integration from 0.5 to 5.0 m increased to mass concentration from 560 to 630 g km^−2^.

### Rise velocities

**Figure 4:**
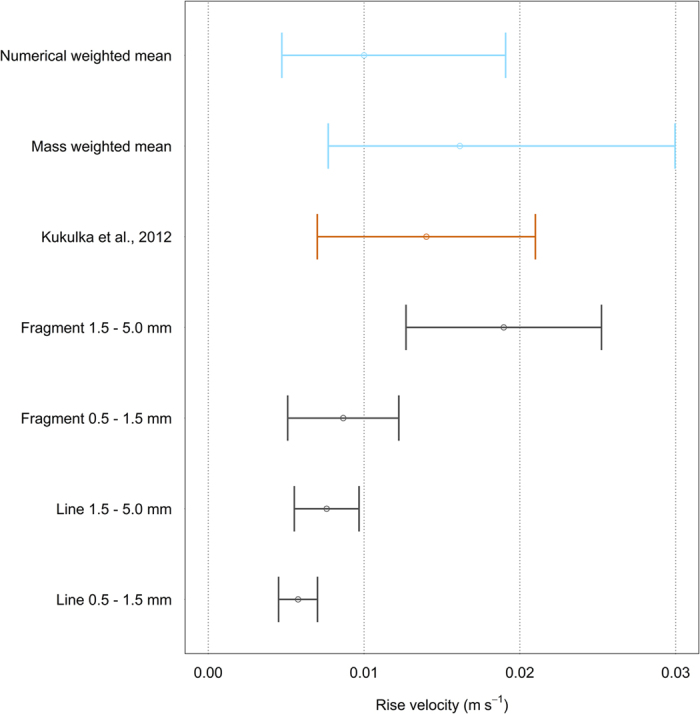
Microplastic terminal rise velocities. Numerical and mass weighted mean terminal rise velocities with weighted minimum and maximum values (blue), the average buoyant terminal rise velocity ± standard deviation reported by Kukulka *et al*.^11^ (orange) and the terminal rise velocities ± standard deviations for different plastic categories (grey).

Rise velocities (average ± standard deviation) for fragments with size 1.5–5.0 mm and 0.5–1.5 mm were 0.019 ± 0.006 m s^−1^ and 0.009 ± 0.004 m s^−1^, respectively. For lines of 1.5–5.0 mm and 0.5–1.5 mm these were 0.008 ± 0.002 m s^−1^ and 0.006 ± 0.001 m s^−1^, respectively (Fig. 4). The buoyant terminal rise velocity values calculated for this study did not differ significantly between fragments 0.5–1.5 mm and lines 1.5–5 mm (p = 0.319, Wilcoxon signed-rank test), but were significantly different (p < 0.05) for all other combinations. Smaller particles had a lower buoyant terminal rise velocity for the same plastic shapes, and for the same size class the buoyant terminal rise velocity of lines was lower compared to fragments.

### Vertical distribution model

**Figure 5:**
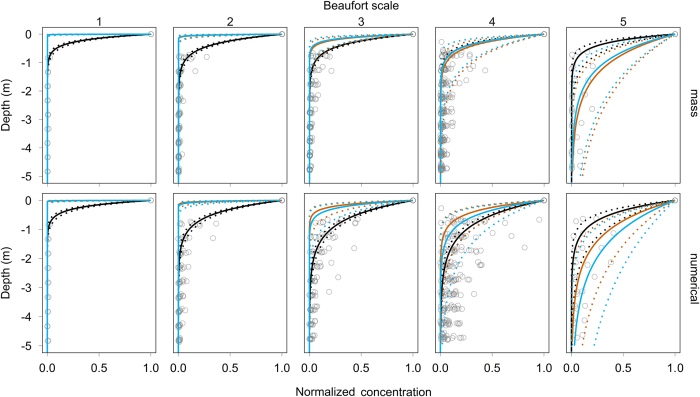
The vertical distribution of buoyant microplastics (0.5–5 mm) in the ocean. Grey circles show normalised mass (top panels) and numerical (bottom panels) microplastic concentrations. The exponential model is shown for three different parameterizations: (1) fitted parameters (black lines), (2) hydrodynamic parameterization with the average buoyant terminal rise velocity reported by Kukulka *et al*.^11^ (orange lines) and (3) hydrodynamic parameterization with the weighted buoyant terminal rise velocity from the experiments of this study (blue lines). Mean parameter values (solid lines) with their uncertainty intervals (dotted lines) are indicated. Concentrations exceeding surface concentrations are not visualised in this figure, but are included in the statistical model.

The exponential decrease model fitted the data well (R^2^ varied between 1.00 and 0.75), with λ values decreasing with higher wind speeds. With depth, mass concentrations decreased more rapidly than numerical concentrations, particularly under higher Beaufort sea states. Results from the hydrodynamic parameterization of the model are shown in Fig. 5, using both the mean ± standard deviation buoyant terminal rise velocity reported by Kukulka *et al*.^11^, as well as the mean, minimum and maximum weighted rise velocities obtained in this study (Fig. 4). The uncertainty intervals of the statistically fitted parameter and the hydrodynamic parameter were compared. These intervals overlapped only for Beaufort 4, for Beaufort 3 when given the mass concentration and the buoyant terminal rise velocity of Kukulka *et al*.^11^ and for Beaufort 5 when using the numerical concentration only. Only for Beaufort 3, using the mass concentration and the buoyant terminal rise velocity of Kukulka *et al*.^11^, did the hydrodynamic mean value fall within the confidence interval of the fitted parameter. For Beaufort 1 and 2, the hydrodynamic parameterized model predicted less mixing compared to the statistically fitted variant, while this was reversed for Beaufort 5. The goodness of fit of the hydrodynamically parameterized model with different parameter values (e.g. buoyant terminal rise velocity, wind speed, wave height) decreased with increasing wind speeds, from 1.0 for Beaufort 1 to 0.11 for Beaufort 5.

**Figure 6:**
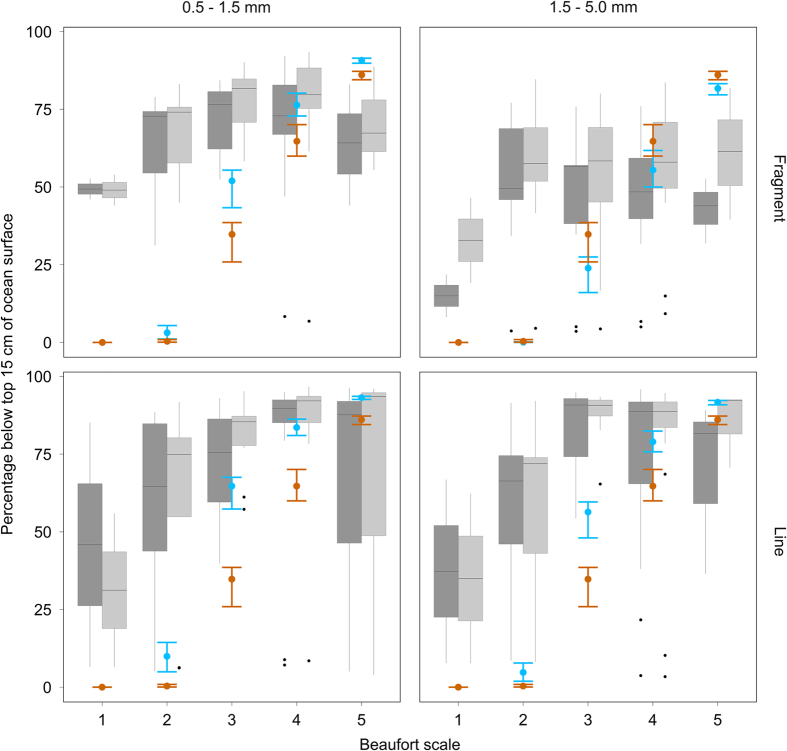
The percentage of microplastics missed by manta trawls. Mass (light grey) and numerical (dark grey) abundance of microplastics missed by manta trawls (0–15 cm sampling depth), for different sea states, plastic particles types and plastic particle sizes. Boxplot outliers are shown as black circles. We also depicted these underestimations (mean ± standard deviation) as predicted by the model of Kukulka *et al*.^11^ when using their as well as our rise velocities (orange and blue, respectively).

Size and type of microplastics significantly explained the differences in the decrease coefficients (p < 0.05), both for mass and numerical results. Based on our statistical fitted parameter λ, we calculated average, minimum and maximum correction factors, which correct surface concentrations for mixing to 5 m depth. Average values were calculated assuming that Beaufort 1–5 occur equally often. For manta nets (sampling depth is 0–15 cm)^30^, microplastic mass concentrations are underestimated by a factor 2.38 (1.04–26.9), and numerical concentrations by a factor 2.74 (1.04–30.0). With a neuston net (sampling depth 0–25 cm^11^), the underestimation factor decreases to 1.85 (1.00–16.2) and 2.08 (1.00–18.0) for microplastic mass and numerical concentrations respectively. An extrapolation of the exponential fit to 17 m, the average mixed layer depth of this study, results in underestimations factors of 2.39 (1.04–57.7) for mass and 2.75 (1.05–80.0) for numerical abundance when sampling with manta nets, and 1.86 (1.00–34.8) for mass and 2.09 (1.00–48.1) for numerical abundance when sampling with neuston nets (Fig. 6). Based on the hydrodynamic parameterization, manta tow underestimate concentrations on average with a factor 1.5 (1.0–6.5) (when using the average buoyant terminal rise velocity reported by Kukulka *et al*.^11^, and with a factor 1.7 (1.0–13.5) when using the rising velocities estimated in this study. In general, the underestimations based on our buoyant terminal rise velocity were greater than those based on the buoyant terminal rise velocity of Kukulka *et al*.^11^. When applying the statistically parameterized model for plastic types and sizes separately, the extent of the underestimations hardly differed between mass and numerical concentrations. Underestimations tended to increase with growing wind speeds, and were more profound for lines than for fragments, especially within the 1.5–5.0 mm size class (Fig. 6).

## Discussion

We studied the vertical mixing of buoyant microplastics (0.5–5 mm), based on extensive data from the North Atlantic accumulation zone. Numerical and mass concentrations decreased exponentially with depth, the strongest decline occurring in calmer oceans and for bigger particles. Similar to previous research^12^,^31^, this study shows that small lines are most susceptible to wind mixing, because of their relatively low buoyant terminal rise velocity. Microplastic mass concentrations decrease more rapidly with depth compared to numerical concentrations, which is caused by the direct relationship between microplastic size/mass and its buoyant terminal rise velocity. The average buoyant terminal rise velocity for fragments of 1.5–5.0 mm was higher than those reported for microplastics in other studies^11^,^12^,^15^. The velocities of the other plastic categories were within the confidence interval of Reisser *et al*.^12^ and on the lower end of the confidence interval reported by Kukulka *et al*.^11^ and Brunner *et al*.^11^,^12^,^15^. On average 60% of the buoyant microplastics present in the first 5 m of the water column are overlooked when sampling with manta or neuston nets, these underestimations ranging between 3.4 and 97%. Underestimations of total microplastic amounts increase towards higher Beaufort scales as well as smaller and more line-shaped particles.

Other studies report underestimation factors in the same order of magnitude as found in our study. Brunner *et al*.^15^ report that surface concentrations are on average a factor 3.7 and 5.3 higher, with maximum of 88 and 79, for the Atlantic and Pacific Ocean respectively^15^. Another study predicted a maximum of 70% underestimation, or a factor 3.3^12^. In this study, surface concentrations are on average a factor 2.6 higher, with a maximum of 30. This correction factor is only representative if Beaufort 1–5 occur in the same frequency. An average correction factor of 3.1 is found when taking the average of all samples, without taking into account how often certain wind speeds occur. As each study measures under different wind conditions, both the averages reported in this study as well as the averages of other studies, should be considered with care.

Both mass and numerical abundance of microplastics are reported in this study, since measurements of mass are required for mass balance and emission studies, while particle numbers are more relevant for the assessment of exposure risks^10^. Higher depth-integrated concentrations were found in this study compared to other North Atlantic field observations^32^,^33^. Microplastic quantification comparisons across different studies must be made with caution as both the mesh size of nets and the sample processing protocol influences the encountered number of microplastics. For instance, one study reported that particle numbers were 100,000 times greater when using an 80 μm mesh net instead of a 450 μm^34^. Numbers reported here are only representative for the 0.5–5.0 mm mesh, and are likely to increase when decreasing the mesh size. When correcting for wind mixing^11^, modelled microplastic mass abundance in the centre of the North Atlantic gyre, has been estimated at 100–800 g km^−2^ 16,17 which is similar to the average of 630 g km^−2^ found in the present study.

Recent studies observed a lower than expected amount of millimetre-sized particles at the surface, when assuming a linear fragmentation model in equilibrium^9^,^16^,^35^. Different vertical transport mechanisms have been suggested to explain this size-selective removal, including sinking (due to ingestion-egestion^16^, marine snow^36^,^37^ and/or biofouling^16^,^38^,^39^,^40^) as well as mixing^11^,^12^,^16^,^20^. Wind mixing has been shown to partly explain the removal of particles from the surface^11^,^12^,^15^. It was found that small lines and fragments are more susceptible to wind mixing, because of their low buoyant terminal rise velocity, which strengthens the hypothesis that wind mixing causes a size selective loss of plastics from the surface. Several studies have corrected their surface measurements for wind mixing^10^,^16^,^17^,^18^,^19^ using the model of Kukulka *et al*.^11^. This model can adequately predict the vertical mixing, although the statistically best fit does not always coincide with the hydrodynamic parameterization of the model. It is likely that the relatively high λ value for Beaufort 5 is caused by a lack of samples towards the higher end of this sea state, and that the hydrodynamically parameterized model is more representative for Beaufort 5. For Beaufort 1 and 2, the statistical parameterization predicts more mixing compared to the hydrodynamic parameterization. For example, the hydrodynamically parameterized model predicts that 100% of the plastic mass is present at the surface (0–15 cm) for Beaufort 1, while the statistically fitted model predicts that of all plastic present in the first 5 m of the water column only 63% is present at the surface. For Beaufort 2, the underestimation of surface trawls is predicted to range between 0 and 10% with the hydrodynamic parameterization, while the statistical fit predicts underestimations of 60% on average. It is unknown which of these two parameterizations predicts the vertical distribution most accurately, because we lack detailed data between 0 and 1 m depth. It is possible that the statistical parameter was overfitted, as there are very few data points present to estimate the steepness of the curve between 0 and 1 m depth. The hydrodynamic parameter was calculated from different components, namely the buoyant terminal rise velocity, the wave height and the frictional water velocity (depending on wind speed). For wave height and the conversion from wind speed to frictional water velocity no uncertainty was included, which could also explain some of the difference. Extensive data for the first meter of the water column, especially for low wind speeds, would help to understand the vertical distribution better.

The hydrodynamic model of Kukulka *et al*.^11^ has often been used to correct microplastic surface samples for wind mixing^10^,^16^,^17^,^18^,^19^, but we show here that these estimations are associated with high levels of uncertainty, with some potential bias (e.g. underestimations for low Beaufort scales). Furthermore, these studies^10^,^16^,^17^,^18^,^19^ used manta or neuston measurements and their associated sea states to estimate depth-integrated concentrations of microplastics, without taking particle size and type differences into account. In this study, we show for the first time that estimations of depth-integrated concentrations of microplastics should be done across different particle sizes and types as these particles have distinct buoyant terminal rise velocity ranges. This is particularly relevant for those attempting to find the ‘missing’ plastic^9^,^16^,^35^. By accounting for these differences we can obtain more realistic buoyant plastic size distributions. Only then it is possible to make a statement as to which proportion of size-selective removal is explained by other processes such as sinking and degradation.

Other vertical microplastic mixing models exist, that focus on Langmuir circulations and breaking waves^15^, surface heat fluxes^20^ or theoretical turbulence processes^41^. For the mixing of microplastics <0.5 mm, Langmuir circulation, breaking waves and heat-induced mixing seem to be key drivers. A study in the North Atlantic calculated that only 4.6 and 1.5% of 10 and 100 μm particles respectively are present in the first meter, compared to 95% for 1 mm particles^41^. All models predict steep concentration decreases within the first 0–3 meters for Beaufort <6, similar to the findings of this study. Most of these models focus on the vertical distribution of microplastics throughout the mixed layer depth, but are based on relatively scarce datasets^15^,^20^. Although microplastics are present throughout the mixed layer, our results as well as several other studies^12^,^15^,^20^,^41^ suggest that the majority of the 0.5–5.0 mm buoyant microplastics is present in the first 0–3 m of the water column. When extrapolating the statistical model to 17 m depth, the increase in microplastic amounts is minimal, indicating most microplastics are present in the upper layer.

Knowledge of the distribution of plastics, both horizontally and vertically, is essential to understand and mitigate their impact on the marine environment^10^. As buoyant microplastics are present close to the ocean surface, they are potentially available for a large range of surface feeders, including endangered vertebrates, neuston and zooplankton species^42^. Not only the abundance, but also plastic characteristics such as shape, size and density are likely affecting the impact on marine life^3^. Plastic input into the marine environment is expected to increase an order of magnitude by 2025 43 and plastic fragmentation processes may not yet have reached a steady state^44^. The extensive dataset on microplastic vertical distribution presented in this study helps to better understand the distribution of microplastics. However, more sampling is needed to gain more extensive knowledge about the extent of this pollutant, focussing on coastal waters, differences in vertical distributions during day and night, sampling towards smaller size classes and including more sea states, both on the low and high end of the Beaufort scale. Overall, general vertical mixing models, applicable to all oceans, covering large ranges of plastic characteristics and vertical mixing processes, are therefore essential to fully understand and mitigate the impact of plastic pollution on the marine environment.

## Additional Information

**How to cite this article**: Kooi, M. *et al*. The effect of particle properties on the depth profile of buoyant plastics in the ocean. *Sci. Rep.* **6**, 33882; doi: 10.1038/srep33882 (2016).
